# Enhancing Documentation Precision in Ophthalmic Surgery: A Quality Improvement Project

**DOI:** 10.7759/cureus.51274

**Published:** 2023-12-29

**Authors:** Shafiq Tanveer, Maryum Khilji, Nazli Gul, Syed Hassnain Shah, Hamza Haroon, Luqman Khan, Asna Tahir, Ahad Nawaz, Ayesha Khilji, Rao E Hassan

**Affiliations:** 1 Ophthalmology, Khyber Teaching Hospital, Peshawar, PAK; 2 Orthopaedics and Trauma, Pakistan Institute of Medical Sciences, Islamabad, PAK; 3 Surgery, Ayub Teaching Hospital, Abbottabad, PAK; 4 Orthopaedics and Trauma, Khyber Teaching Hospital, Peshawar, PAK; 5 Accident and Emergency, District Headquarters Teaching Hospital, Dera Ismail Khan, PAK; 6 Internal Medicine, Hayatabad Medical Complex, Peshawar, PAK

**Keywords:** memory aids, continuous medical education (cme), documentation errors, quality improvement projects, surgery, ophthalmology, audit

## Abstract

Background

Effective clinical documentation, particularly operative notes, is essential for maintaining healthcare standards and fostering interdisciplinary communication. This study focuses on improving the quality of ophthalmic operative notes by adopting the Royal College of Surgeons (RCS) guidelines for good surgical practice.

Methodology

A retrospective cross-sectional audit at Khyber Teaching Hospital, Pakistan, assessed 138 operative notes against the RCS criteria. After an educational session and the placement of memory aids in operation theaters, a re-audit of 125 notes was conducted. Parameters were selectively applied based on relevance to specific cases, and omissions were discussed with the local ethical committee.

Results

The initial audit revealed deficiencies in 10 critical areas, with only three parameters exceeding 85% accuracy. The re-audit showed significant improvement across these parameters, achieving documentation of 85.3% of all criteria. Paired t-test results indicated a substantial difference in documentation quality before and after interventions.

Conclusions

A combined strategy involving surgeon education, memory aids, and adherence to established standards significantly enhances operative note quality. The study underscores the importance of sustained reinforcement mechanisms for continuous improvements in documentation practices.

## Introduction

Effective clinical documentation is crucial for maintaining high healthcare standards, with operative notes being a key component for interdisciplinary communication [1]. Comprehensive records play a crucial role in improving patient care and advancing medical research, ensuring long-term patient follow-up [2].

Operative notes provide healthcare practitioners with profound insights into procedure specifics and postoperative care [3]. Surgeons heavily rely on these detailed records to make pivotal decisions tailored to individual patient cases, emphasizing the indispensable nature of maintaining flawless note quality for refined surgical patient management. Moreover, it assumes a crucial role as a medicolegal document in addressing and resolving inconsistencies or conflicts [4].

Faced with the absence of specific directives for documenting ophthalmic operations, we chose to adopt the Royal College of Surgeons (RCS) guidelines for good surgical practice, which outlines 18 criteria for achieving the highest documentation standards [5].

In this study, we propose enhancing operative note quality via thorough auditing processes and targeted educational sessions. This involves incorporating the criteria established by the RCS guidelines.

## Materials and methods

This cross-sectional retrospective audit was conducted at the Ophthalmology Department of Khyber Teaching Hospital, Peshawar, Pakistan. Approval from the Khyber Medical College Peshawar Institutional Research and Ethical Review Board (IREB) was secured in May 2023 (approval number: 737/DME/KMC). The primary objective was to assess the quality of operative notes within the tertiary care facility against the standards of RCS [5]. The goal was to identify missing details, lack of completeness, and variations, requiring necessary modifications.

In May 2023, an evaluation of 138 operation notes from procedures conducted between March 15th and April 30th, 2023, was done as an initial audit. Compliance with the criteria outlined in the RCS good surgical practice guidelines was ensured. After the initial audit, a training session disseminated results, promoting comprehensive discussions on RCS guidelines. A memory aid with guideline parameters was placed in operating theaters. In November 2023, a re-audit covered 125 operation notes from procedures performed between October 1st and November 15th, 2023. Every operative note was documented using a handwritten proforma and completed by trainees or attending ophthalmologists. Outcomes were recorded on a digital checklist.

It was acknowledged that not all criteria could be fulfilled in a single operation. Broad assessments were conducted by selectively applying parameters defined by the RCS based on their relevance to specific cases. Criteria deemed irrelevant were marked as “not applicable” on the checklist. Both audits involved a comprehensive evaluation. For instance, in cataract surgery, the “identification of any prosthesis used” criterion involved verifying the documentation of the implanted lens along with its serial number. Details of closure, including suture material and technique, were meticulously expected for all surgeries. All ophthalmic operations were included in the study without exclusion criteria based on procedure type. Moreover, two parameters given in the RCS guidelines, namely, “anticipated blood loss” and “deep vein thrombosis prophylaxis” were omitted from the study because they were considered irrelevant to the operative procedures being performed in this setting. These omissions were discussed with the local ethical committee beforehand and their approval was sought. A total of 17 parameters were assessed and comprised the variables in this study.

After the second data collection, results were presented to the local audit committee, expressing contentment with observed improvements, completing the audit cycle. Data analysis was done using SPSS Statistics for Windows, version 26.0 (IBM Corp., Armonk, NY, USA), employing a paired t-test, with a p-value <0.05 being significant.

## Results

A thorough examination was conducted on 263 operation notes, with 138 from the initial audit and 125 from the subsequent re-audit.

**Figure 1:**
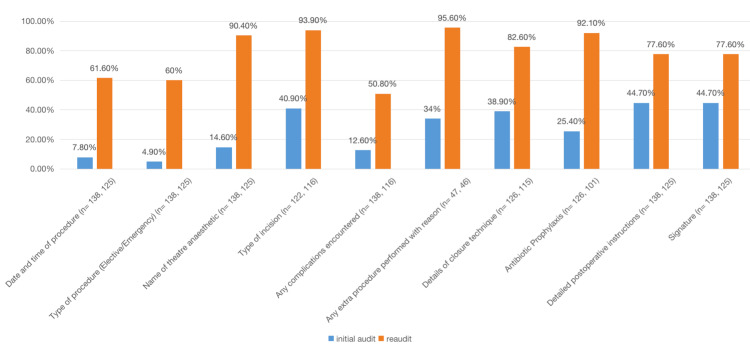
Comparison between documentation frequency of 10 parameters. The data are represented as percentages (%); n: total number of operation notes applicable for a specific parameter. Paired t-test; p-values for all the parameters given in this figure were <0.05.

In the initial audit, only three parameters were identified with more than 85% accuracy. Deficiencies were observed in 10 critical areas, i.e., documenting the date and time of the procedure (n = 11, 7.8%), categorizing the procedure as emergency or elective (n = 7, 4.9%), recording the name of the theater anesthetist (n = 20, 14.6%), specifying the type of incision (n = 50, 40.9%), documenting encountered complications (n = 17, 12.6%), detailing additional procedures with reasons (n = 16, 34%), describing closure technique details (n = 49, 38.9%), prescribing antibiotic prophylaxis (n = 32, 25.4%), providing detailed postoperative instructions (n = 62, 44.7%), and signing the operation sheet (n = 62, 44.7%). In the subsequent re-audit, a significant improvement was noted across these 10 parameters (53.8%, 55.1%, 75.8%, 53%, 38.2%, 61.6%, 43.7%, 66.7%, 32.9%, and 32.9%, respectively). A paired t-test was used for the analysis of individual parameters, and the p-value of these 10 parameters was <0.05 (Figure 1).

In summary, the initial audit demonstrated documentation of 48.6% (standard deviation (SD) = 30.6) of all parameters in operative notes. The re-audit demonstrated substantial improvement, achieving documentation of 85.3% (SD = 15) of all parameters, signifying a cumulative increase of 36.7% from the initial audit (Table 1). The results of the paired t-test decisively indicated a significant difference in the quality of documentation in operative notes before and after the training session, along with the placement of memory aids in the operating theater (mean = 48.6, SD = 30.6 versus mean = 85.3, SD = 15), with a p-value <0.05.

**Table 1:** Compliance with the documentation of parameters in both the initial audit and the re-audit. The data has been represented as percentage (%); n: Total number of operation notes applicable for a specific parameter Paired t-test; p-value <0.05 significant

Parameter assessed	Compliance in the initial audit (n)	Compliance in the re-audit (n)	Difference	P-value
Date and time of procedure	7.8% (138)	61.6% (125)	53.8%	<0.05
Type of procedure (elective/emergency)	4.9% (138)	60% (125)	55.1%	<0.05
Name of surgeon	99% (138)	100% (125)	1%	>0.05
Name of assisting surgeon	74.8% (138)	98.4% (125)	23.6%	<0.05
Name of theater anesthetist	14.6% (138)	90.4% (125)	75.8%	<0.05
Name of the operative procedure	97.1% (138)	96.8% (125)	-0.03%	>0.05
Type of incision	40.9% (122)	93.9% (116)	53%	<0.05
Operative diagnosis	70.9% (138)	96.8% (125)	25.9%	<0.05
Operative findings	68.9% (138)	89.6% (125)	20.7%	<0.05
Any complications encountered	12.6% (138)	50.8% (116)	38.2%	<0.05
Any extra procedure performed with reason	34% (47)	95.6% (46)	61.6%	<0.05
Details of tissue removed, altered, or added	61.3% (111)	98% (100)	36.7%	<0.05
Details of closure technique	38.9% (126)	82.6% (115)	43.7%	<0.05
Antibiotic prophylaxis	25.4% (126)	92.1% (101)	66.7	<0.05
Identification of any prosthesis used	85% (87)	88.7% (80)	3.7%	>0.05
Detailed postoperative instructions	44.7% (138)	77.6% (125)	32.9%	<0.05
Signature	44.7% (138)	77.6% (125)	32.9%	<0.05

## Discussion

Ensuring precise surgical notes of ophthalmic surgery is crucial for effective patient management and upholding quality care standards. Despite its pivotal role, this aspect has received insufficient attention in existing textbooks and literature. The RCS guidelines, published in 2014, meticulously outlined 18 criteria essential for writing a comprehensive surgical operation note [5]. Previous studies have evaluated operation note quality based on RCS standards, proposing diverse methodologies to enhance overall quality [6]. In the context of our investigation, a noteworthy enhancement in the quality of operation notes was brought with surgeons’ education and the implementation of memory aids. The findings underscore the positive impact of educational initiatives and memory support tools on the precision and comprehensiveness of surgical notes, thereby adding important ideas to the overall discussion about improving the way operation notes are documented.

**Figure 2:**
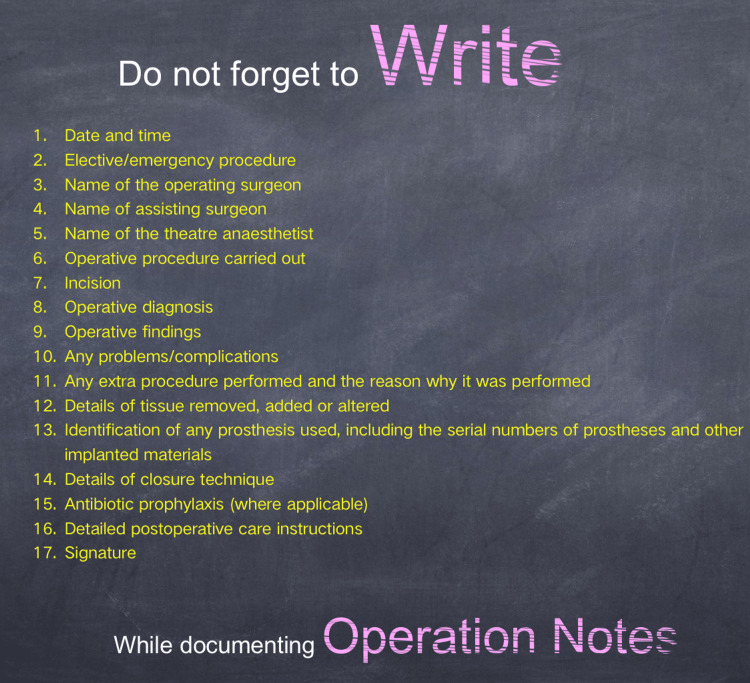
A memory aid placed in operating theaters to aid surgeons in the documentation of operative notes.

The significance of proformas in optimizing operation notes is well-established [7,8]. Despite having a well-structured proforma for documenting operative notes in our hospital, the initial audit revealed deficiencies in 10 parameters outlined by the RCS guidelines. Consistent with earlier research, this study confirmed the common shortcomings in documenting operation notes [9,10]. In response, we conducted an educational session and introduced a memory aid containing guideline parameters in the operating theaters (Figure 2). Following a reasonable adaptation period, the second audit demonstrated a substantial improvement in the identified 10 parameters. This methodology aligns with the work by Din et al., who investigated orthopedic operation notes covering both elective and emergent procedures. Their study resulted in a significant improvement, crediting the implementation of a memory aid for a notable increase in overall documentation accuracy, rising from 90% to 97.1% [11]. These collective findings, fortified by our institution’s internal audit, underscore the transformative impact of a straightforward intervention, integrating a memory aid within the operation theatre, on enhancing the quality of documentation. Following this model, Bateman et al. extended the application of memory aids into the theaters of otolaryngology. In their subsequent re-audit, each part of the operation notes exhibited a remarkable and comprehensive improvement [12].

Adjusting established habits, particularly in surgical documentation, presents unique challenges. Surgeons commonly overlook certain details in operation notes, which are instead recorded in alternative forms by key members of the surgical team, such as the operating room nurse or anesthetist [10]. Critical information such as antibiotic prophylaxis and surgery duration is usually documented by anesthetists on intraoperative charts. However, for improved clarity and accessibility, it is strongly recommended to directly incorporate these details into the operation notes. This practice ensures comprehensive information for all involved in patient care, fostering smoother communication and facilitating more informed decision-making processes.

The research consistently supports the preference for computer-based templates or typed notes over handwritten ones [13]. Our hospital has yet to adopt electronic operation notes due to financial limitations. There is a strong conviction that electronic note documentation establishes elevated standards for completeness in operative procedures [14]. Furthermore, the implementation of standardized templates for operation notes, tailored to specific surgical procedures such as ophthalmology, would notably enhance the quality and consistency of documentation.

The importance of utilizing clear and comprehensive notes to prevent postoperative delays and errors is evident [10,15]. Assessing the potential correlation between improved operation note quality and enhanced patient outcomes presents a significant challenge commonly faced by studies aiming to elevate documentation standards. Further research is crucial to show the impact of well-documented surgical notes on patient outcomes.

This study has some limitations that necessitate careful consideration. First, the generalizability of the findings may be hindered due to potential variations in the effectiveness of interventions across diverse healthcare settings, surgical specialties, and varying levels of medical expertise. Additionally, as the study did not capture the long-term effects of educational initiatives and memory aids, this raises concerns about potential oversights regarding sustained improvements in documentation practices, underscoring the importance of implementing consistent and ongoing reinforcement mechanisms, such as recurrent educational sessions, to ensure continued positive impacts on operative note documentation.

Through the examination of current practices, this audit not only identifies opportunities for improving surgeons’ documentation but also highlights the positive impact of aligning practices with established standards. The audit cycle consistently demonstrated that adherence to these standards and subsequent re-audits led to substantial improvement. Evident from the work of Bozbiyik et al., integrating memory aids and educational initiatives shows more promising results, especially when implemented alongside a well-structured proforma [16]. In summary, the study emphasizes the effectiveness of a combined strategy, incorporating education and the use of a memory aid, to optimize documentation practices.

## Conclusions

The study provides valuable insights into optimizing surgical documentation practices, highlighting the transformative impact of a combined strategy involving surgeon education and memory aids. The findings emphasize the significance of sustained reinforcement mechanisms for continuous positive impacts on operative note documentation.
